# Biofunctional prosthetic system: A new era complete denture

**DOI:** 10.4103/0975-7406.76505

**Published:** 2011

**Authors:** Vandana Saini, Ruchi Singla

**Affiliations:** Faculty of Dentistry, Dr. H.S.J. Dental College, Punjab University, Chandigarh, India. E-mail: teena2982@yahoo.co.in

Sir,

This clinical report describes the prosthetic rehabilitation of an edentulous patient,who was dissatisfied from her 8-year-old denture. To give her a better fit, we opted Biofunctional Prosthetic System (BPS) for the new prosthesis. BPS is the system designed to work with the body in a biologically harmonious way, maximizing function, and giving comfort and natural appearance to the patient. The functional impression technique and simulation of the jaw movements by the Stratos 200 articulator in BPS ensure that BPS denture meets most exacting requirements.[1]

BPS denture meets the esthetic demand of patients with its unique Ivoclear teeth, which replicate anatomy of the natural tooth Ivoclear teeth are made up of 3 layers of cross-linked acrylic resins that contribute to a life-like appearance and resistance to wearing. BPS system uses a controlled heat/pressure polymerization procedure during which time the exact amount of material flows into the flask to compensate for shrinkage, which ensures a perfect fit. This pressure also optimizes the physical properties of the denture. [2]

**Figure 1:**
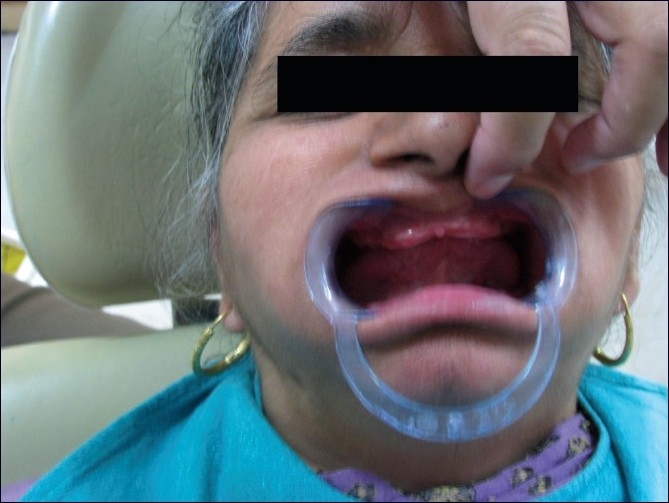
Resorbed ridges

A 60-year-old edentulous woman with a chief complaint of compromised function and esthetics was treated in the clinic. Intraoral examination showed resorbed ridges and masticatory dysfunction [Figure 1]. An extraoral examination revealed flattened mandibular plane. She was wearing dentures with attrited teeth and worn out denture base. A significant loss of vertical dimension affected the temporomandibular joint. Hence, a BPS denture was planned to give her a better fitted prosthesis.

**Figure 2:**
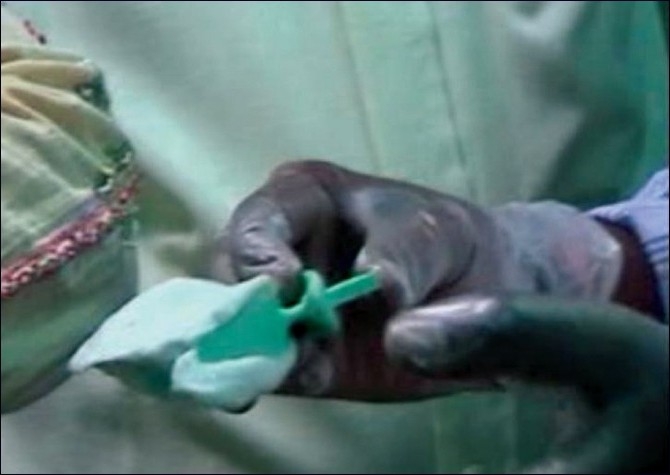
Occlussal centric tray loaded with impression for recording initial vertical dimension

**Figure 3:**
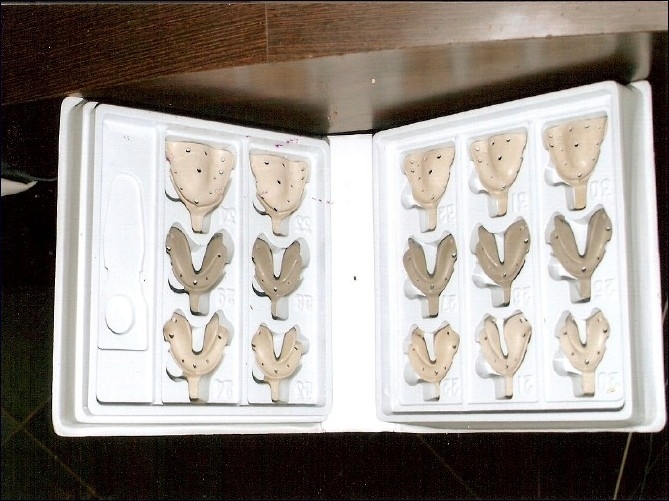
Biofunctional prosthetic system impression trays

**Figure 4:**
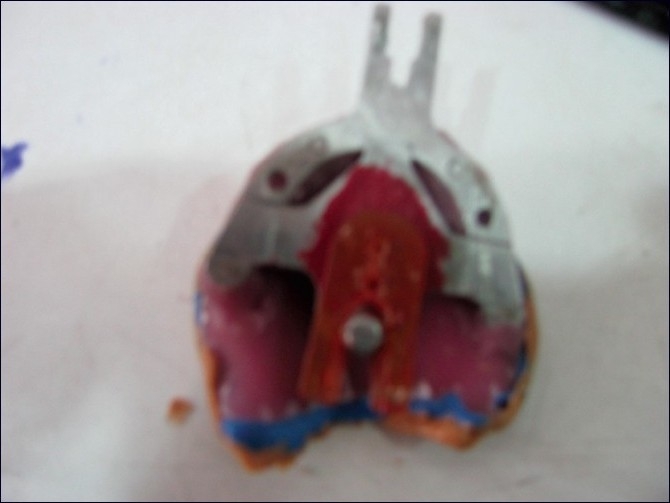
Bite registration through Gnathometer M

The BPS recommends impression making similar in principle to the mucostatic method that minimally compresses tissues, using a combination of irreversible hydrocolloids of varying densities together in the same impression.[3] Low-density impression material (syringe Acc Gel) was syringed into the vestibular area and the occlusal centric tray was loaded with high-density hydrocolloid and inserted into the patient’s mouth to get the initial vertical dimension [Figure 2]. This vertical dimension was used for mounting the casts obtained from initial impressions, taken with Accu-trays (different from conventional denture trays) with an extra flange to cover the vestibular areas and extended distal part to cover the retromandibular pad area more efficiently [Figure 3]. Custom trays were made on the primary casts. The Gnathometer M tracing device was attached to the casts, which facilitates the clinical procedures of secondary impression making, face-bow record and jaw registration [Figure 4].

**Figure 5:**
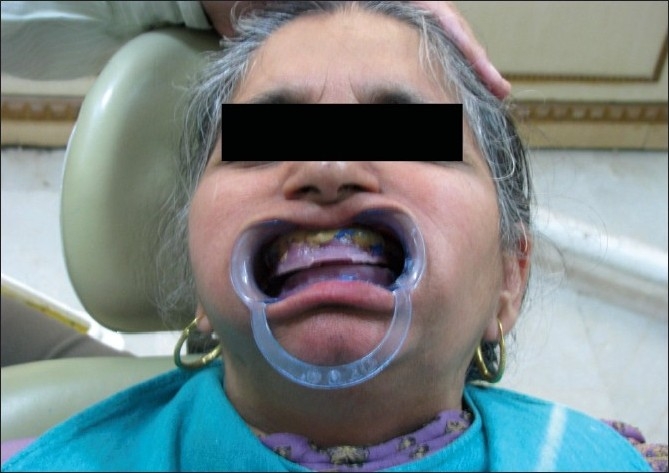
Secondary impression-making with zinc oxide eugenol paste

**Figure 6:**
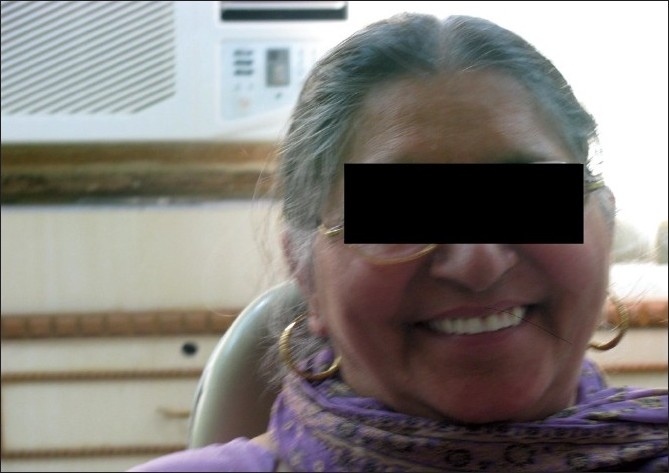
Wax-up trial for the patient

**Figure 7:**
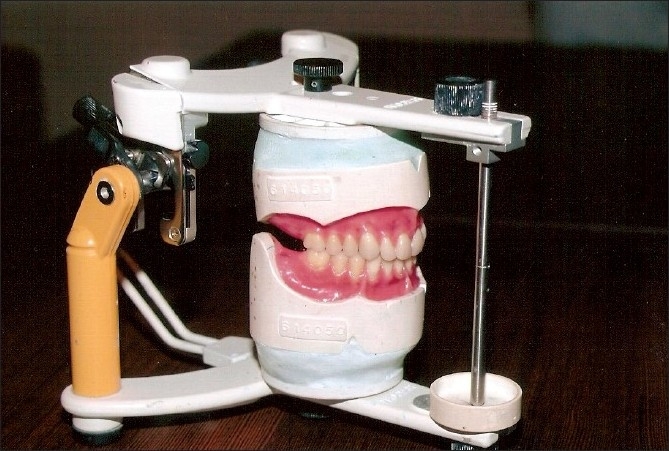
Acrylized denture

The secondary impression was taken with zinc oxide eugenol impression paste [Figure 5]. Casts were poured and a wax-up denture was made for the trial [Figure 6]. After checking the fit and occlussal relations, the denture was sent to the laboratory. Dentures were cured with injection molding technique [Figure 7] using Ivocap high-impact plus denture base material.[4] Necessary adjustments were done and the dentures were delivered to the patient.

The patient was recalled after 6 months and examined. There was no occlusal disharmony or sore spots. The patient was very much satisfied with her new prosthesis and she showed her gratification for the comfortable prosthesis and a younger look.
